# Antibacterial Characteristics of Nanoclay-Infused Cavit Temporary Filling Material: In Vitro Study

**DOI:** 10.3390/jfb16080299

**Published:** 2025-08-19

**Authors:** Bahareh Nazemi Salman, Ayda Notash, Ali Ramazani, Shaghayegh Niaz, Seyed Mohammadrasoul Naeimi, Shayan Darvish, Ionut Luchian

**Affiliations:** 1 Department of Pediatric Dentistry, School of Dentistry, Zanjan University of Medical Sciences, Zanjan 4513956184, Iran; drnazemi@zums.ac.ir; 2 School of Dentistry, Tabriz University of Medical Sciences, Tabriz 5178654714, Iran; aidantsh78@gmail.com; 3 Department of Pharmaceutical Biotechnology, School of Pharmacy, Zanjan University of Medical Sciences, Zanjan 4513956184, Iran; ramazania@zums.ac.ir; 4 Independent Researcher, Urmia 5719175546, Iran; dr.shaghayeghniyazi1999@gmail.com; 5 School of Dentistry, Zanjan University of Medical Sciences, Zanjan 4513956184, Iran; emadnaeiminasir@gmail.com; 6 Independent Researcher, Tucson, AZ 85741, USA; 7 Department of Periodontology, Faculty of Dental Medicine, “Grigore T. Popa” University of Medicine and Pharmacy, Str. Universitatii 16, 700115 Iasi, Romania; ionut.luchian@umfiasi.ro

**Keywords:** antibacterial, nanoclay, nanoparticles, temporary dental filling

## Abstract

In pediatric endodontic procedures, final crown placement is often delayed, requiring the use of temporary filling materials to seal the access cavity. Given the importance of antibacterial properties in temporary restorations and the known antimicrobial effects of nanoclay particles, this study aimed to evaluate the antibacterial efficacy of a nanoclay-infused temporary dressing against cariogenic and residual intracanal bacteria. A commercial temporary material (CAVISOL, Tehran, Iran) was blended with nanoclay (SOUTHERN, Gonzalez, TX, USA; average size 95 nm), using eugenol as a wetting agent. The antibacterial effects on *Streptococcus mutans*, *Enterococcus faecalis*, and *Escherichia coli* were assessed using disc diffusion, well diffusion, and microtiter plate assays after 24 h of incubation at 37 °C (six material groups, three bacterial strains, three replications for each antibacterial test). Statistical analyses were performed using Shapiro–Wilk and ANOVA tests (*p* < 0.05). The results showed that formulations containing 60% and 80% nanoclay significantly inhibited the growth of all tested bacteria, outperforming pure Cavit (*p* < 0.05). The most substantial inhibition was observed in *E. coli*, while *S. mutans* exhibited the least susceptibility (*p* < 0.05). These findings suggest that incorporating nanoclay into temporary fillings may enhance efficacy to increase the success rate of pediatric endodontic treatments, although further physicochemical and clinical evaluations are warranted.

## 1. Introduction

Success in treatments like pulpotomy and pulpectomy is closely related to the complete elimination of microorganisms from the root canals through proper biomechanical cleaning. Effective disinfection and sealing of the access cavity with a restorative material are key to treatment success [1]. Physical cleaning significantly reduces bacterial count, but due to the anatomical complexity of root canals, especially in deciduous teeth, complete microorganism elimination may not be achievable in treatments like pulpotomy and pulpectomy [2]. To maintain the aseptic chain, coronal sealing during treatment sessions is crucial, preventing saliva and microorganisms from infiltrating the cleaned canals. Studies indicate a direct relationship between coronal sealing and treatment success. Therefore, temporary filling materials used for access cavities should have antibacterial properties and maintain this feature over time [3]. Currently, in many dental procedures such as root canal treatment, final crown restoration does not occur in the first session, necessitating the temporary filling of the access cavity. A temporary filling material is recognized as a restorative that remains inside the access cavity until the final restoration is completed. However, microleakage of filling materials may lead to bacterial contamination in the canal and periapical complications in the post-treatment period. Studies indicate that materials based on zinc oxide have superior antibacterial properties compared to materials based on resin or calcium hydroxide [4,5]. The most widely used zinc oxide-containing material among multi-session temporary filling materials is Cavit [6]. Between treatment sessions, Cavit provides specific advantages such as resisting bacterial contamination, preventing saliva leakage, and preventing the fracture of thin tooth walls [7]. According to surveys, the use of this material is convenient both for filling cavities and for easy removal before final restoration [8]. However, studies by Brännström and colleagues have shown that Cavit lacks proper sealing ability, leading to bacterial contamination in the cavity [9]. Torabinejad and colleagues demonstrated that this bacterial infiltration can reach the apical area within 48 h [10]. Cavit temporary fillings can demonstrate an antimicrobial effect against coronal leakage for a maximum of 7 days [1]. Therefore, adding antibacterial materials to temporary fillings allows the maintenance of a bacteria-free environment, even in cases of partial bacterial infiltration from the coronal access cavity, and can contribute to strengthening the coronal seal, preventing the proliferation of remaining microorganisms in inaccessible areas [11]. Studies on oral bacteria, such as *Streptococcus mutans, Escherichia coli*, and *Enterococcus faecalis*, have identified them as common causes of dental caries and periodontal diseases [12,13]. We chose *Escherichia coli* as a model Gram-negative organism to demonstrate the material spectrum. We will clarify this rationale and consider testing more clinically relevant species such as *Lactobacillus* or *Actinomyces* in future studies. Moreover, there is also a significant association between acute dental caries and chronic diseases affecting the growth of children [14]. Given the escalating bacterial resistance, there is a crucial need to identify viable alternatives to conventional antibacterial methods. In the field of dentistry, the emergence of nanotechnology has garnered significant interest, as antibacterial agents derived from nanomaterials offer advantages such as cost effectiveness, stable structures, exceptional antibacterial properties, and a wide-ranging antibacterial spectrum [15]. Nanotechnology involves imaging, modifying, and modeling functions on a nanometer scale. Nanoparticles, which are smaller than 100 nanometers, exhibit unique properties such as high surface activity, chemical reactivity, and biological activity [16].

Nanoclays are nanoparticles that are used as antibacterial agents in dental materials [17]. Montmorillonites (MMT) are a type of nanoclay that has gained significant research interest in recent years, with layered structures that are used extensively in polymers to enhance their mechanical properties [18,19,20]. Incorporating only a small amount of nanoclay into polymers improves their mechanical performance [21]. The use of montmorillonite nanoparticles is an effective strategy for formulating new dental materials with clinical applicability [22,23]. Even adding 2% weight of quaternary ammonium-modified montmorillonite nanoparticles increases the antibacterial properties of temporary restorative materials [24]. Studies have reported that nanoclay is non-toxic to the human body and exhibits the highest antimicrobial property without side effects at low concentrations [25]. While the antibacterial property of temporary restorations is crucial for treatment success, studies have shown that commercially available temporary restorations often lack this property [11].

This study is aiming to assess the hypothesis that incorporating nanoclay into Cavit will enhance its antibacterial properties against common oral microorganisms, including *Streptococcus mutans*, *Escherichia coli*, and *Enterococcus faecalis*.

## 2. Materials and Methods

This in vitro experimental study was conducted in a controlled environment, ensuring aseptic conditions. 

### 2.1. Materials and Bacteria

All tests were conducted on standard strains of *Enterococcus faecalis* ATCC 29212, *Streptococcus mutans* PTCC 1683, and *Escherichia coli* ATCC 25922, obtained from the microbiology department of Zanjan University of Medical Sciences. The temporary filling material used in this study was CAVISOL, obtained from Golchai Iran Pharmaceutical Company (Tehran, Iran). The nanoclay, designated as 5A, was acquired from the Southern Company, Atlanta, GA, USA. The average particle size was 59 nanometers, with an interlayer spacing of 2.7 nanometers.

### 2.2. Experimental Groups

To prepare the experimental groups, the following materials were mixed in the specified proportions. Eugenol drops were added to achieve a paste-like consistency and ensure a homogeneous mixture of the temporary filling material. The study consisted of 6 different groups:

1. Cavit1 (C1): negative control = 100% by weight CAVISOL temporary filling material + 3 drops eugenol.
2. Cavit2 (C2): 80% by weight CAVISOL temporary filling material + 20% by weight nanoclay + 3 drops eugenol.
3. Cavit3 (C3): 60% by weight CAVISOL temporary filling material + 40% by weight nanoclay + 3 drops eugenol.
4. Cavit4 (C4): 40% by weight CAVISOL temporary filling material + 60% by weight nanoclay + 3 drops eugenol.
5. Cavit5 (C5): 20% by weight CAVISOL temporary filling material + 80% by weight nanoclay + 3 drops eugenol.
6. Cavit6 (C6): positive control = 100% by weight nanoclay + 3 drops eugenol.

### 2.3. Tests

In this study, the following antibacterial tests were conducted:

1. Zone of Inhibition Testing: the zone of inhibition was evaluated using two methods, disk diffusion and the well diffusion assay.
2. The microtiter dish assay.

### 2.4. Implementation Method

#### 2.4.1. Preparation of Experimental Groups

The temporary filling material, Cavisol (Tehran, Iran, Golchai), was physically mixed with nanoclay powder (Closite 5A, Gonzalez, TX, USA, Southern) with particle sizes averaging 59 nanometers using a digital balance (Ohaus Pro Scout SP601, Parsippany, NJ USA) to ensure precise weight measurement. The mixture was prepared with weight ratios of 0.8 to 0.2, 0.6 to 0.4, 0.4 to 0.6, and 0.2 to 0.8, respectively.

#### 2.4.2. Preparation of Culture Media

In this study, MUELLER-HINTON AGAR, BLOOD AGAR, and BROTH media from Merck Germany were used. These powders were mixed with distilled water according to the manufacturer’s instructions, poured into Erlen Meyer flasks, and then boiled. The flasks containing the culture media were then placed in an autoclave under standard conditions to be sterilized. After the autoclaving process was completed, the flasks containing the culture media were removed from the autoclave and, next to the flame, AGAR was poured into plates, and BROTH was poured into test tubes. After solidification of the mentioned media, plates containing culture media were placed in an incubator for 24 h to detect any potential contamination during their preparation.

#### 2.4.3. Preparation of Bacterial Suspension

Standard bacterial strains, lyophilized in ampoules, were prepared by following aseptic standards and microbiological methods, washed, and then used to prepare a suspension. These suspensions were introduced into Moller–Hinton agar culture media and incubated to promote the bacterial growth of *Enterococcus faecalis* and *Escherichia coli*. For *Streptococcus mutans* bacteria, BLOOD AGAR culture media was used. To ensure uniform colonies of bacteria, the McFarland 0.5 standard was employed using a BROTH culture.

#### 2.4.4. Disc Diffusion Test

Antibiotic discs with a diameter of 6 mm were prepared and immersed in the experimental groups (Cavit 1–6). A yellow sampler was dipped into a microbial suspension with a 0.5 McFarland concentration, and 200 microliters of the microbial solution was poured onto a 10 cm agar plate. Bacteria were cultured on the surface of the plate using the spread plate method. Discs soaked in the prepared compositions (Cavit 1–6) were placed on the plate. A disc without immersion in any mixture was used as a negative control. After 24 h of incubation at 37 degrees Celsius, the plates were examined to evaluate the growth inhibition halo diameter, measured in millimeters using a caliper. Moreover, the plate containing Streptococcus mutans was placed in a CO_2_ incubator to create anaerobic growth conditions. This procedure was performed for all three bacteria, and the testing process was repeated three times for each bacterium.

For each method, we used 6 material groups × 3 bacterial strains × 3 replicates = 54 samples; the total sample size was 162.

This approach is consistent with previous in vitro antimicrobial studies, where triplicate testing per group is considered standard and sufficient for statistical analyses such as one-way ANOVA or Kruskal–Wallis tests [26,27].

#### 2.4.5. Well Diffusion Test

In this method, initially, under sterile conditions next to the flame and within the laminar flow hood, 200 microliters of the 0.5 McFarland bacterial suspension was spread on the surface of the plate containing Muller–Hinton agar (blood agar for *Streptococcus mutans*) using the spread plate method. Subsequently, five wells were created with a diameter of 5 mm, spaced at 2 cm intervals on the plate’s surface. Then, 0.15 g of each of the compounds from groups 1–6 was placed in its own well. In this test, group 6 served as the positive control, and group 1 was the negative control. All plates were incubated at 37 degrees Celsius for 24 h. After this incubation period, bacterial cultures were examined for the presence or absence of growth inhibition zones, and the diameter of these zones was measured in millimeters using a caliper. This test was repeated three times [28].

#### 2.4.6. Biofilm Assessment Test (Microtiter Dish Assay)

Aqueous extracts from temporary filling materials were prepared using groups 1–6, each with distinct weight percentages, and their impact on the formation of biofilm by *Enterococcus faecalis*, *Streptococcus mutans*, and *Escherichia coli* was assessed [29].

This test was conducted in multiple stages:

- Preparation of PBS Solution:

To prepare one liter of PBS solution, 8 g of NaCl, 0.2 g of KCl, 0.2 g of Na_2_HPO_4_, and 0.24 g of KH_2_PO_4_ were dissolved completely in 800 milliliters of distilled water. The pH was adjusted to 7.4 using sodium hydroxide (NaOH) solution with a pH meter, and the final volume reached one liter.

- Preparation of Extracts from Groups 1–6:

A 0.2 g sample of each composition was placed inside labeled glass vials for groups 1–6, and 1 milliliter of PBS solution was added to each vial. The vials were shaken slightly and incubated for 10 h in a shaking incubator at 37 degrees Celsius and 81 rpm. This process was repeated for each group. The extracts were then transferred to labeled microtubes. In each well of a 24-well plate, 150 microliters of the extract was added using a blue sampler. Next, 150 microliters of PBS was added to two wells and they functioned as the control groups. The following procedure was performed for the three bacteria using three separate plates.

- The cultivation of seven bacterial strains in broth culture medium and the preparation of 0.5 McFarland concentration.
- Treatment with extracts from temporary filling materials and nanoclay: in each well containing the extract, 50 microliters of the bacterial suspension with a concentration of 0.5 McFarland was dispensed using a yellow sampler.
- Incubation: incubation was performed for 24 h, with the plates containing *Escherichia coli* and *Streptococcus mutans* placed in an incubator at 37 degrees Celsius, and the plates containing *Enterococcus faecalis* placed in a CO_2_ incubator at 37 degrees Celsius.
- Fluorescent biofilm staining: After incubation, the contents of the plates were carefully aspirated using a sterile pipette. To detach non-adherent bacteria, each well was washed three times with 200 microliters of sterile PBS. Subsequently, in each well, 200 microliters of 4% crystal violet was added. After 12 min, the excess dye was poured off, and the plates were washed under a gentle stream of distilled water.
- Recording biofilm absorption using the ELISA plate reader (Tecan EL-Reader, Männedorf, Switzerland): After washing the crystal violet, 200 microliters of 33% acetic acid solution was added to each well for 15 min. At this stage, the colors that had penetrated the biofilm were released, and then they were read using the ELISA instrument at a wavelength of 600 nanometers. The more bacterial biofilm remained, the more color it absorbed. Therefore, after adding acetic acid, it produced more color, ultimately registering a higher absorption in the ELISA instrument.

## 3. Results

### 3.1. Well Diffusion Test

**Figure 1:**
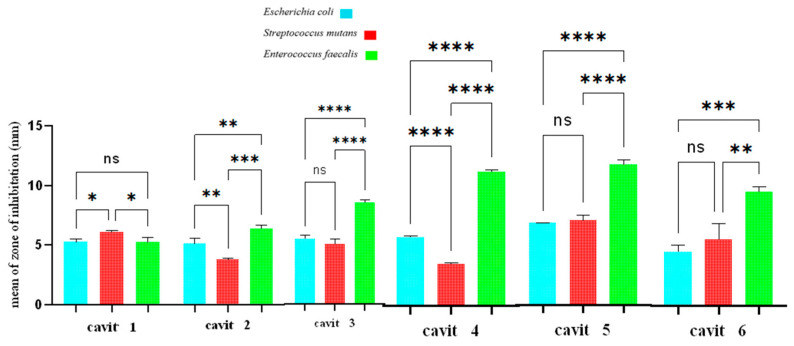
Comparison of inhibition zones in well diffusion test. ns: not significant, *: *p*-value < 0.05, **: *p*-value < 0.01, ***: *p*-value < 0.001, ****: *p*-value < 0.0001.

**Figure 2:**
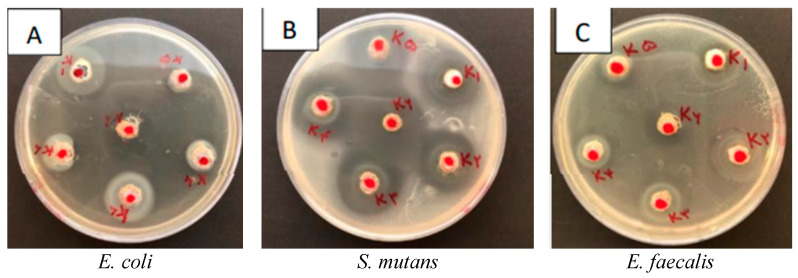
Inhibition zones of each bacterium in well diffusion test. (**A**) *E. coli*, (**B**) *S. mutans* (**C**) *E. faecalis*.

The findings are depicted in Figure 1, with the individual results for each bacterium provided in Table 1. A Picture of the inhibition zones is provided in Figure 2.

### 3.2. Disk Diffusion Test

**Figure 3:**
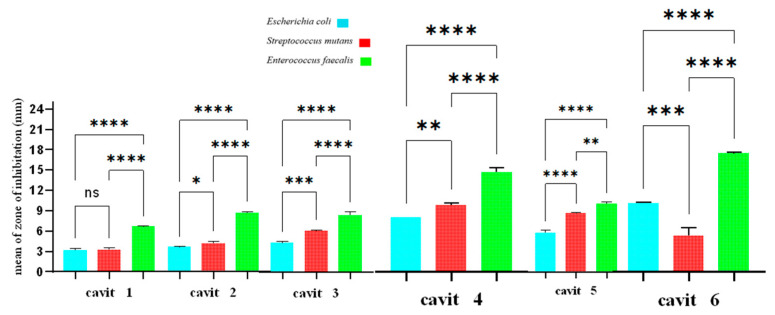
Comparison of inhibition zones in disk diffusion test. ns: not significant, *: *p*-value < 0.05, **: *p*-value < 0.01, ***: *p*-value < 0.001, ****: *p*-value < 0.0001.

The findings are depicted in Figure 3, with the individual results for each bacterium provided in Table 2.

In disk and well diffusion assays, an increased diameter of the inhibition zone reflects higher inhibitory efficacy against the tested microorganisms (Table 1 and Table 2).

For *E. coli*, Cavit-3 in the well diffusion test and Cavit-6 in the disk diffusion test showed significantly greater inhibition than the other groups. For *S. mutans*, Cavit-5 demonstrated markedly superior inhibition compared to the other groups in both the well diffusion test and the disk diffusion test. For *E. faecalis*, Cavit-5 in the well diffusion test and Cavit-6 in the disk diffusion test achieved a markedly enhanced inhibitory performance over the other groups (Figure 1 and Figure 3).

The findings from both the disk and well diffusion assays demonstrate that the antibacterial activity of the Cavit–nanoclay formulations differs depending on the bacterial species tested. The results suggest that *E. coli* is more susceptible to formulations with higher nanoclay content, whereas *S. mutans* responds best to Cavit-5, and *E. faecalis* shows moderate sensitivity across different formulations. This variability highlights the need to consider bacterial species-specific responses when designing antimicrobial dental materials, to ensure maximum efficacy against targeted pathogens.

### 3.3. Microtiter Dish

**Figure 4:**
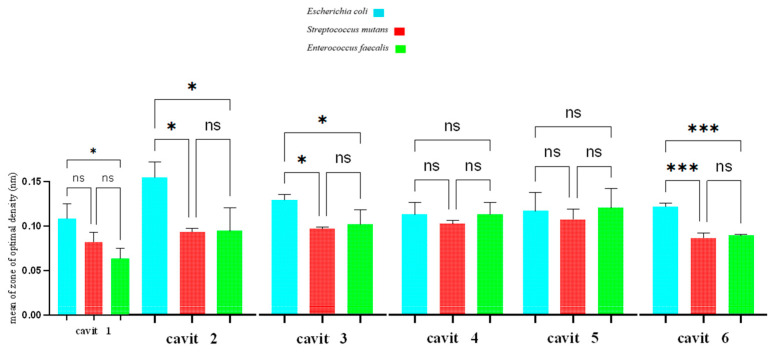
Comparison of inhibition zones in microtiter dish assay test. ns: not significant, *: *p*-value < 0.05, ***: *p*-value < 0.001.

The findings are depicted in Figure 4, with the individual results for each bacterium provided in Table 3.

In this test, smaller values denote higher inhibitory efficacy against the tested microorganisms (Table 3).

For *E. coli*, Cavit-4 showed significantly greater inhibition than Cavit-3 and Cavit-2 (*p* < 0.001). For *S. mutans*, Cavit-6 produced the largest inhibition zone, with no significant differences among the other groups. For *E. faecalis*, no statistically significant differences were observed among the tested groups.

Overall, these results indicate that the antibacterial effect of the materials varies by bacterial species, with *E. coli* being more sensitive to higher nanoclay concentrations, while *S. mutans* responded most strongly to Cavit-6, and *E. faecalis* showed comparable resistance across formulations.

## 4. Discussion

We demonstrated that incorporating nanoclay Cloisite 5A particles into Cavit can augment its antibacterial characteristics. The following sections will elaborate on the material weight percentage that exhibits the most significant antibacterial impact on each bacterium.

*E. faecalis*: In the well diffusion method, the Cavit5 group (20 wt% Cavit + 80 wt% nanoclay + 3 drops eugenol) demonstrated the highest antibacterial efficacy, and in the disk diffusion method, the Cavit6 group (100 wt% nanoclay + 3 drops eugenol) did the same. Moreover, all groups showed statistically significant differences in inhibiting bacterial growth. This finding aligns with the research conducted by Alzaidy et al., where they observed an augmentation in the antibacterial characteristics of root canal sealers against *Enterococcus faecalis* upon the addition of silver nanoparticles [30].

*E. coli*: In the well diffusion method, the Cavit3 group (60 wt% Cavit + 40 wt% nanoclay + 3 drops eugenol) exhibited the largest zone of *Escherichia coli* growth inhibition, and in the disk diffusion method, the Cavit6 group (100 wt% nanoclay + 3 drops eugenol) did the same, although its difference was not statistically significant (*p*-value = 0.05).

*S. mutans*: The Cavit5 group (20 wt% Cavit + 80 wt% nanoclay + 3 drops eugenol) showed the highest antibacterial properties for Streptococcus mutans in both the disk diffusion and well diffusion methods. The study titled “Nanoclay-reinforced polymethylmethacrylate and its mechanical properties” demonstrates that even modest nanoclay additions (1–2 wt%) can significantly reduce the tensile strength of PMMA resin. Therefore, incorporating nanoclay at levels as high as 80% is not considered clinically feasible and should be regarded as applicable only in the context of in vitro microbiological testing [31].

Consistent with the current study, Yin et al. showcased the enhanced antibacterial properties of dental materials through the addition of silver nanoparticles [27]. Similarly, Fareed et al. found that incorporating 2% weight of nanoclay improves antibacterial and physical properties, supporting our results [32].

However, Barzegar et al.’s study, using 20A Closite nanoclay, contradicts our findings. They could not statistically demonstrate enhanced antibacterial and mechanical properties in polymethyl methacrylate materials combined with nanoclay [31]. The difference likely arises from the nanoclay type (5A Closite) and variations in nanoparticle size. Particle size and morphology significantly influence antibacterial effects [33]. Organically modified nanoclays show higher antibacterial activity than unmodified forms due to increased surface reactivity [34]. Smaller particles and better dispersion typically enhance efficacy [35]. Changes in nanoparticle size and surface-to-volume ratio impact distribution and biocompatibility, as extensively studied and confirmed [36]. Our study consistently observed an increase in the diameter of the inhibition zones during the well diffusion and disk diffusion tests with a rise in the percentage of nanoclay. This rise can be attributed to the antimicrobial and bacteriostatic effects of nanoclay. Additionally, Song et al. demonstrated the applicability of nanoparticles due to their small size and high surface-to-mass ratio, which are highly suitable for enhancing antimicrobial effects [37]. The antibacterial mechanism of nanoclay involves interacting with the cell wall and peptidoglycan membrane, resulting in cell lysis, disrupting bacterial proteins and protein synthesis, and interacting with bacterial DNA to hinder DNA replication. As the percentage of nanoclay rises, the antibacterial mechanism intensifies, enhancing antibacterial properties [38].

Among the three examined bacteria, *Escherichia coli* exhibited an increase in antibacterial properties proportional to the increase in the percentage of nanoclay. This implies that the antibacterial properties of the experimental groups, from Cavit1 to Cavit5, consistently increased in *Escherichia coli*. The groups containing 60% and 80% nanoclay showed excellent antibacterial results against *Escherichia coli*. These findings align with the results of the study by Ghorbanpour et al. [38]. The lower sensitivity to the other two bacteria, *Enterococcus faecalis* and *Streptococcus mutans*, can be attributed to their Gram-positive nature. Studies have shown that Gram-positive bacteria, with their thick cell walls, exhibit greater resistance to nanoparticles compared to Gram-negative bacteria such as *Escherichia coli* [39]. Nevertheless, pairwise comparison of the bacteria indicated that among the three bacteria, *Enterococcus faecalis* showed the largest growth inhibition zone. Regarding this contradiction between the results, it should be noted that the type of numerical comparison used for the diameter of the growth inhibition zones may not be accurate, as the growth of each bacterium in a culture medium with another bacterium differs. Therefore, the consistent enhancement of antibacterial properties with an increase in the weight percentage of nanoclay serves as a more reliable criterion for comparing bacterial growth inhibition. This consistent pattern was observed in *Escherichia coli* with both the well diffusion and disk diffusion methods. The results of the microtiter dish assay lacked consistency and did not exhibit a clear pattern in terms of antibacterial properties. One possible explanation is the dissolving of the mixtures (Cavit1 to Cavit6) in PBS. These materials contained eugenol, which is hydrophobic [40]. Thosar et al. investigated the solubility of various materials, including eugenol, and found that eugenol exhibited the least solubility among the materials studied [41]. Although Cavit contains eugenol, its antimicrobial activity is known to be limited and inconsistent, particularly against resistant species such as *E. faecalis*. Previous studies have shown that eugenol exhibits weak antibacterial properties when used in zinc oxide-based pastes [42]. In the present study, significant inhibition zones were only observed with increased nanoclay concentrations, supporting the conclusion that the observed antibacterial enhancement is primarily due to nanoclay rather than eugenol. Considering that PBS is hydrophilic, the dissolution process may have been disrupted. In this study, the well diffusion method proved to be the most promising and reliable approach among the antibacterial tests for evaluating dental materials in combination with nanoparticles. This in vitro study investigated the antibacterial effects of nanoclay in combination with Cavit temporary filling material, acknowledging the different conditions in the oral cavity. Factors like oral temperature, pH, and saliva flow can influence antimicrobial efficacy. Recent reports using talc-mediated nanoclay composites show nearly complete bacterial kill rates with acceptable cell viability (~84%) in vitro, suggesting that antibacterial efficacy can be enhanced without excessive cytotoxicity if phase and surface chemistry are optimized [43]. Future studies should broaden the evaluation of nanoclay’s effects on various oral bacteria, assess its cytotoxic effects on human cells, and thoroughly examine the mechanical properties of this new material.

## 5. Conclusions

This study demonstrates that the incorporation of nanoclay particles significantly enhances the antibacterial properties of Cavit temporary filling material. Among the tested concentrations, higher nanoclay loadings demonstrated superior antibacterial efficacy in vitro; however, further investigation is required to identify the optimal concentration that balances antimicrobial activity, mechanical performance, and cytocompatibility. Although broad clinical application cannot yet be inferred, these findings underscore the necessity for comprehensive in vivo studies and rigorous biocompatibility assessments. Establishing a safe and effective nanoclay concentration may enable its integration as a targeted antimicrobial additive in restorative dentistry. Future studies should also address the long-term stability and potential cytotoxicity of nanoclay-based formulations prior to clinical translation.

## Figures and Tables

**Table 1:** Hierarchy of bacterium inhibition zones in well diffusion test, from most to least in diameter (left to right). Cavity groups are listed based on inhibition zones, from largest to smallest.

***Bacterial* Species **	**Cavity Groups**					
*E. coli*	Cavit3>	Cavit5>	Cavit2>	Cavit4>	Cavit1>	Cavit6
*S. mutans*	Cavit5>	Cavit3=	Cavit1	Cavit6>	Cavit2>	Cavit4
*E. faecalis*	Cavit5>	Cavit3>	Cavit4>	Cavit6>	Cavit2>	Cavit1

**Table 2:** Hierarchy of bacterial species inhibition zones during disk diffusion test. Cavity groups are listed based on inhibition zones, from largest to smallest.

*Bacterial* Species	Cavity Groups					
*E. coli*	Cavit6>	Cavit5>	Cavit4>	Cavit3>	Cavit2>	Cavit1
*S. mutans*	Cavit5>	Cavit4>	Cavit3>	Cavit2>	Cavit6>	Cavit1
*E. faecalis*	Cavit6>	Cavit5>	Cavit4>	Cavit2>	Cavit3>	Cavit1

**Table 3:** Antibacterial effect on each bacterial species during microtiter dish assay. Cavity groups are listed based on inhibition zones, from largest to smallest.

*Bacterial* Species	Cavity Groups					
*E. coli*	Cavit4>	Cavit5>	Cavit6>	Cavit3>	Cavit2>	Cavit1
*S. mutans*	Cavit6>	Cavit2>	Cavit3>	Cavit4>	Cavit5>	Cavit1
*E. faecalis*	Cavit6>	Cavit1>	Cavit2>	Cavit3>	Cavit4>	Cavit5

## Data Availability

The corresponding author can provide data upon reasonable request.
